# Proteomic Applications in Aquatic Environment Studies

**DOI:** 10.3390/proteomes10030032

**Published:** 2022-09-01

**Authors:** Nadeeka Thushari Gajahin Gamage, Rina Miyashita, Kazutaka Takahashi, Shuichi Asakawa, Jayan Duminda Mahesh Senevirathna

**Affiliations:** 1 Department of Aquatic Bioscience, Graduate School of Agricultural and Life Sciences, The University of Tokyo, Tokyo 113-8657, Japan; 2 Department of Animal Science, Faculty of Animal Science and Export Agriculture, Uva Wellassa University, Badulla 90000, Sri Lanka

**Keywords:** aquatic proteomics, biomarkers, ecotoxicoproteomics, protein fingerprinting, multiomics

## Abstract

Genome determines the unique individualities of organisms; however, proteins play significant roles in the generation of the colorful life forms below water. Aquatic systems are usually complex and multifaceted and can take on unique modifications and adaptations to environmental changes by altering proteins at the cellular level. Proteomics is an essential strategy for exploring aquatic ecosystems due to the diverse involvement of proteins, proteoforms, and their complexity in basic and advanced cellular functions. Proteomics can expedite the analysis of molecular mechanisms underlying biological processes in an aquatic environment. Previous proteomic studies on aquatic environments have mainly focused on pollution assessments, ecotoxicology, their role in the food industry, and extraction and identification of natural products. Aquatic protein biomarkers have been comprehensively reported and are currently extensively applied in the pharmaceutical and medical industries. Cellular- and molecular-level responses of organisms can be used as indicators of environmental changes and stresses. Conversely, environmental changes are expedient in predicting aquatic health and productivity, which are crucial for ecosystem management and conservation. Recent advances in proteomics have contributed to the development of sustainable aquaculture, seafood safety, and high aquatic food production. Proteomic approaches have expanded to other aspects of the aquatic environment, such as protein fingerprinting for species identification. In this review, we encapsulated current proteomic applications and evaluated the potential strengths, weaknesses, opportunities, and threats of proteomics for future aquatic environmental studies. The review identifies both pros and cons of aquatic proteomics and projects potential challenges and recommendations. We postulate that proteomics is an emerging, powerful, and integrated omics approach for aquatic environmental studies.

## 1. Introduction

The aquatic environment accounts for over 70% of the Earth’s land surface, with over 95% occurring as seawater. This extensive ecosystem comprises numerous biological, chemical, and physical energy resources. Aquatic proteins from fish, invertebrates, seaweeds, and algae largely contribute to human dietary requirements. Some protein discoveries from aquatic environments with special functions have been used in biotechnological studies for many decades. An example is the identification of green fluorescent protein in aquatic invertebrates by Shimomura in 1962, which is used as a protein marker in various biological studies [1]. Similarly, numerous proteins have been isolated and characterized from aquatic organisms and are used in various industries such as food, pharmaceutical, medical, and environmental monitoring. Key examples are aquatic proteins with repetitive motifs (e.g., the matrix proteins found in pearl oysters) with promising biomedical and biotechnological applications [2]. Aquatic organisms have adapted to various environmental changes. These adaptations could identify by using the diversity of proteoforms, which derives from a single gene within the context of aquatic evolutionary adaptations.

Pioneer protein-based research, which involved their modification through the addition of pentose sugar and phosphates after translation from a DNA fragment [3], led to various development opportunities. Subsequently, proteins became valuable resources contributing to the global economy, and for years, numerous industries have targeted protein-related research fields [4]. Numerous biotechnological companies have, in the past two decades, focused on expanding proteomic applications to solve various biological problems. Applying omics technologies, including genomics, transcriptomics, proteomics, and metabolomics in various studies on aquatic ecosystems, have been widely elaborated. Proteomic application has been reviewed in the field of mobile genetic elements research on microorganisms during their early development stage [5]. Protein markers were also applied in an ecotoxicogenomic study to determine hormonal changes in aquatic organisms because of various environmental contaminants [6]. Ecotoxicoproteomics studies are used to highlight the molecular responses of organisms to environmental stress to achieve the grand concept, “make our planet great again” [7]. Environmental proteomics can be applied to microorganisms and vertebrates; therefore, it is possible to study the long-term adverse effects of environmental toxins on humans and animals [8]. Proteins present in bones preserved in water offer excellent opportunities for forensic studies on aquatic environments. A previous study identified that fetuin-A protein could significantly be deamidated in pond water, and it is used as an important potential biomarker for estimating postmortem submergence time in criminal investigations [9]. Accordingly, the identification of similar protein biomarkers in aquatic environments would accelerate forensic investigations in the future. The interest in aquatic proteomics for various purposes is growing; however, its novel applications in addressing various challenges in the aquatic environment are still lacking due to knowledge and technological gaps.

For years, various studies have investigated the link between functions of aquatic ecosystems and biodiversity [10]. A previous report noted that incorporating aquatic ecosystems in the economic valuation of developing countries can result in remarkable changes in decision-making processes [11]. Furthermore, the link between microbial activities, their habitats, the beneficial role of biofilm formation, or their capacity to control ecosystem deterioration and their contribution to human well-being has comprehensively been studied in different environments [12]. However, the potential application of proteomics in the aquatic studies mentioned above is still in the initial stages. A recent study suggested the incorporation of socioecological, eco-evolutionary, and novel ecosystem dynamics in the ecosystem models as components of the current global challenges to meeting human requirements [13]. Aquatic environmental studies show that most effects on the capacity of ecosystem resilience and vulnerability are caused by physical, chemical, and biological pollution/stressors. The roles of dominant periphyton communities, such as cyanobacteria, and diatoms have been extensively explored as bioindicators for monitoring the aquatic ecosystems [14]. Recent advances in molecular biomarkers have been used to monitor the domestic, industrial, commercial, and agricultural chemical pollution and to broadly assess their potential human health risk implications [15]. Several stress-related proteins, such as protective proteins and chaperones, have recently been identified in terrestrial plants [16]. Hence, these potential biomarkers can be tested in aquatic environmental stress studies. Novel scientific research approaches for the conservation and management of aquatic ecosystems are advanced aquatic environmental science disciplines. Governance, economic, and social perspectives act as powerful marine conservation tools, which can be integrated with research to protect aquatic ecosystems [17].

Assessment of aquatic resources ensures that the status, exploitation level, diversity, and population dynamics are maintained. Basic concepts of stock assessment, processes, population dynamics, and fish stock assessment models can be applied to manage freshwater and marine fish resources using validated data collection techniques [18]. For commercial aquatic resources, novel technological approaches with value addition to fish and fishery or aquatic products are important to ensure safety and diversification. Aquatic byproducts, waste, and unutilized biomass can be converted into value-added beneficial byproducts with broad-spectrum applications because of their functional properties, diversified nutrient pool, and biopolymer composition [19]. Consequently, this review critically evaluates the broad scope applicability of proteomics as an advanced and reliable tool for aquatic environmental studies.

The aim of this review is to critically evaluate the potential applications and recent advances of proteomics in various aquatic environmental studies, its challenges, and future opportunities. A general overview of proteomics and multiomics applications will help elucidate their novel opportunities in the aquatic sector. One of the significant applications of aquatic proteomics is its promises to achieve sustainable development goals (SDGs). Those goals can be achieved by applying proteomics to aquatic food production and value addition, environment conservation and management, marine pharmaceuticals as well as medicine, and aquatic bioengineering. Additionally, aquatic environment and organism relationships, homeostasis, and metabolism can be revealed by integration of proteomics with other omics technologies, such as genomics, transcriptomics, and metabolomics.

## 2. Methods: Search and Selection of Literature

**Figure 1:**
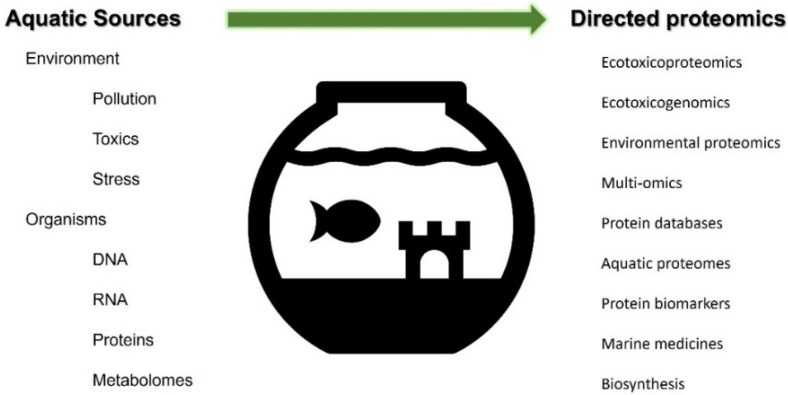
Keywords related to aquatic proteomics studies. The literature survey was conducted while identifying the related terms of aquatic sources, organisms, and proteomics.

From the available literature, three relevant fields that integrate aquatic proteomic approaches were initially identified, including the aquatic food sector (I), aquatic environmental pollution and monitoring (II), and natural aquatic proteins and marine medicine (III). Subsequently, online search engines, such as Google Scholar and PubMed, were utilized using various relevant keyword combinations to retrieve published proteomic information in each selected field. The search keywords in different fields were as follows: in the aquatic food sector, words such as aquaculture proteomics, seafood proteomics, fish proteomics, fishery proteomics, and seaweed proteomics were searched; in the aquatic environmental pollution and monitoring category, searched words included environmental proteomics, ecotoxicogenomics, ecotoxicoproteomics, pollution proteomics, and conservation proteomics; and in the aquatic natural protein and marine medicine category, marine natural products, aquatic drug proteomics, aquatic protein biomarkers, aquatic bioactive compounds, and marine protein biosynthesis were searched. Overlapping keywords, including aquatic environment, aquatic proteomics, aquatic proteome, aquatic multiomics, and aquatic proteins, were used to enhance relevant information search (Figure 1). Approximately 100 published articles were retrieved from indexed and nonindexed peer-reviewed journals and are used in this review. The literature survey facilitated the identification of current trends in proteomics, gaps, limitations, and future opportunities associated with aquatic environmental studies.

## 3. Proteomics as a Biomonitoring Tool

All biological forms consist of a diverse array of proteins essential for their various functions. Proteomics identifies and studies total proteins in each tissue or cell [20]. Proteins are active molecules in an organism and are usually modified post-transcriptionally into different isoforms after mRNA synthesis from DNA fragments. Compared with nucleotides, protein diversity is very high, and a single gene can form more than 100 different proteins [21]. Thus, proteins have been used as monitoring tools in various sectors, such as medicine [22,23], forensic [24], aquaculture [25,26], environmental monitoring [27], and agriculture [28].

Significant progress has been made in proteomics, such as protein extraction and purification, quantification, characterization, sequence structure, and bioinformatic analysis [29]. Sample preparation and protein extraction are critical steps in proteomic studies. Consequently, novel protein extraction kits and protocols have been developed for various plant and animal tissues or cells. An ideal protein extraction protocol and kit should be simple, cost-effective, efficient, and rapid [30,31]. Currently, no universal protein extraction protocol is available for any biological sample from the aquatic environment [32]. A study on heart proteomics revealed that the in-solution digestion shotgun method was better than on-filter digestion or on-pellet digestion isolation methods for characterizing proteins from a dynamic range of tissues in a simple, inexpensive, straightforward, fast, and robust manner [33]. This technique could therefore be used to extract proteins in samples obtained from evolutionary-related aquatic organisms such as marine mammals. A study on aquatic animals showed that protocols, such as the TRIzol method, were suitable for protein extraction from the gill of *Mytilus galloprovincialis* and liver of *Paralichthys olivaceus*, whereas the trichloroacetic acid–acetone solvent method was better in extracting proteins from the soft tissue of *Nereis diversicolor* [34]. A combination of laboratory-made lysis buffers with sonication, followed by protein quantification by either bicinchoninic acid or Lowry assays, and silver staining has been suggested as the best protein extraction or characterization protocol in foraminifera [35]. Selecting or designing suitable buffers for protein isolation is critical, as they could easily damage proteins. Therefore, several aspects of laboratory-made buffers, including pH, buffering system, salts, and reducing and stabilizing agents have been investigated [36]. Some marine organisms, such as seaweed (*Palmaria palmata*), have high polysaccharide levels that require pretreatment to effectively extract proteins [37].

Proteomic analysis can be first categorized as gel-based and gel-free. The label-based and label-free strategies are both gel-free and mass spectrometry (MS)-based techniques. The gel-based technique is used for global protein separation and quantification. An older, two-dimensional gel electrophoresis (2D-GE) technique is popularly used; however, it exhibits some major limitations, including loss of all membrane proteins, the appearance of multiple proteins in a single spot, and the occurrence of a single protein in multiple spots. Consequently, MS techniques have become popular as the most reliable protein separation and quantification alternatives [38]. Label-based protein quantification methods require a stable labeling isotope for peptides, whereas label-free methods can determine the relative or absolute protein quantity using MS techniques. We speculate that integration of the use of 2D-GE with MS-based strategies (both label-based and label-free) could provide more reliable results with high-quality data for further proteomics analysis. However, such methods might still have the same limitations and drawbacks.

Liquid chromatography–mass spectrometry (LC–MS) is suggested as one of the most suitable novel techniques for protein characterization, with a wide use in proteomics because it overcomes major limitations of previous methods and can easily be automated. Common chemical label-based techniques include isotope-coded affinity tags, isotope-coded protein labeling, and isobaric tags for relative and absolute quantification (iTRAQ). The iTRAQ tags are mainly designed for peptides, and their reagents are extremely sensitive. Hence, the iTRAQ technique is more relevant for large-scale, bottom-up proteomics analysis of organism responses in aquatic environments. Tandem mass tag (TMT) labeling is also a label-based method that has been used to identify more than 5000 proteins in sea cucumbers [39] and can also be considered to be a suitable technique for comparative aquatic proteomic studies. Another study noted that the matrix-assisted laser desorption ionization–time-of-flight mass spectrometry (MALDI–TOF MS) is a less expensive and simple method for characterizing aquatic bacteria using protein extractions [40,41]. The MALDI–TOF technique has several applications in aquatic proteomics [42] and it shows great potentiality because the analyses are easy, rapid, robust, high-throughput, and cost-effective. It could represent an interesting alternative to traditional methods of identification of aquatic microorganisms/species despite the high initial cost of the mass spectrometer.

Protein extraction buffers might isolate a unique set of proteins with different hydrophobicity, pI (isoelectric point), aromaticity, and molecular weight. Therefore, this information might help develop enzyme-linked immunosorbent assay (ELISA) methods for assessing seafood allergies [32]. A recent study has developed a protocol for metagenomics applications to isolate proteins and DNA and to digest or extract peptides from microbial biomass in seawater [43]. Marine-based protein hydrolysates are common in food and fish feed composite; therefore, a standard protocol has been developed to characterize the abundance and diversity of their protein complexes, for example, in shrimp, tuna, krill, squid, tilapia, and salmon [44]. For decades, advanced technologies have been used to isolate and identify proteins from organisms. Assessments of protein structure similarities and differences are difficult because of their complexities that require keen bioinformatic analysis to correctly perform fingerprinting [45]. Recent advancements, such as dynamic structural vibration assessments, have been applied in protein fingerprinting (or peptide mass fingerprinting) [46]. However, structural vibrations are used as alternative analytical techniques for protein identification that cleave proteins into smaller peptides; then, their mass is measured and analyzed via mass spectrometry. Additionally, protein–protein interaction studies are essential for elucidating changes in metabolic pathways of aquatic organisms caused due to environmental stress. A recent study used protein fingerprinting to identify the intercellular protein mechanisms of H_2_O_2_ stress-mediated inhibition on algae, *Scenedesmus obliquus* [47].

Scientists have constructed protein databases to enhance protein identification and explore proteoforms complexity. Currently, UniProt is the most updated protein database (https://www.uniprot.org/, accessed on 27 January 2022), comprising two platforms, including Swiss-prot, with 565,928 reviewed data points by experts, and TrEMBL, which has 225,013,025 computationally generated data points. Pfam is another protein database that contains a total of 19,632 protein families and millions of sequences (http://pfam.xfam.org/, accessed on 27 January 2022). Protein Blast or BLASTP is another strategy for retrieving reliable, highly homologous protein sequences for the identification of unknown or new proteins (https://blast.ncbi.nlm.nih.gov/Blast.cgi, accessed on 27 January 2022). For structural analysis, 3D protein shapes can be predicted by several tools, such as the AlfaFold protein structure database (https://alphafold.ebi.ac.uk/, accessed on 2 February 2022), and Protein Data Bank (https://www.rcsb.org/, accessed on 2 February 2022). Additionally, Expasy is an online platform for various protein analyses and related studies (https://www.expasy.org/, accessed on 2 February 2022). For aquatic studies, a fish proteomic database has been developed and used for biomarker discovery (http://www.cifri.res.in/Fishprot/, accessed on 22 February 2022). Similarly, numerous protein data and analytical tools are currently available due to the rapid technological development, bioinformatics, and advancement in artificial intelligence. Therefore, proteomics is a promising biomonitoring tool that will offer numerous novel proteomics-based study opportunities in the aquatic environment.

## 4. Applications of Proteomics in Aquatic Studies

We identified three basic areas with unique proteomic applications in aquatic science, including the aquatic food industry, aquatic environmental monitoring, and aquatic natural product development and future trajectories. Although the above sectors involve a range of aquatic organisms from algae to marine mammals, common isolation techniques are used in their protein isolation and characterization. Additionally, the genomes of many aquatic species are yet to be completed; thus, final omics prediction studies on aquatic life still need improvements.

### 4.1. Proteomics in the Aquatic Food Industry

Aquatic foods largely contribute to the global food chain, and their diversity ranges from microorganisms to mammals. Proteins are basic nutritional components in seafood, and a wide variety of aquatic foods provides numerous opportunities for the development of various products [48]. According to a recent publication, aquatic animal source food consumption is expected to increase by 2030, whereas terrestrial animal meat consumption will reduce due to malnutrition, diet-related diseases [49], and cultural or religious perspectives.

Aquaculture is one of the sustainable approaches to acquiring valuable nutrients, such as eicosapentaenoic acid and docosahexaenoic acid from aquatic foods. Various types of fish, invertebrates (mainly shellfish), plants, and algae are well-known for their role in developing novel aquaculture practices. Contemporary aquaculture farming is currently undergoing growth to improve nutrient availability and sustainability [50]. Genomic information is available for some aquaculture fish species, and identifying causal genes is important for future aquaculture development [51]. Genomes of many cultured species are currently available because of the development of NGS technologies. Functional genomic approaches are essential for understanding gene and peptide functions. Therefore, the identification of active genes, proteins, and their expression dynamics are basic steps used in advanced proteomic techniques.

However, genomics and proteomics are still crucial for the discovery of novel aquaculture species and for assessing their development in the aquaculture system. Consequently, the past two decades have witnessed a significant increase in the application of proteomics to investigate aquatic species and their products. Proteomics is a powerful tool for addressing numerous challenges related to welfare, nutrition, health, production, safety, and quality in aquaculture systems [26]. Targeted and discovery proteomics are two critical approaches applied to enhance the welfare, health condition, nutritional composition, and well-being of cultured fish [52].

The rate of fish growth is a critical factor that affects aquaculture yield, and integrated proteomic techniques can be used to modify metabolic networks and pathways associated with fish growth. Proteomic techniques can also be applied to identify the relationship between genome and protein abundance for growth improvement. A previous report indicated that the incorporation of β-glucan in feed enhanced the growth efficiency and immunity of some fish species [53]. For example, β-glucan dietary supplementation for the rainbow trout, *Oncorhynchus mykiss*, resulted in increased levels of tropomyosin isoforms and a decrease in heavy- and light-chain isoforms of fish fillet myosin [54]. Another aspect is the development of sustainable, eco-friendly culture media for fish farming, since excess feed materials are potential environmental pollutants. Proper feed waste management can be achieved by proteomic techniques for identifying feed waste-degrading enzymes or microbes that can clean the culture environment.

The efficiency of culture feeding could be enhanced through the use of nutritionally rich, low-cost feed formulations, which require low feeding cycles. In Salmonids, incorporating vegetable oil in feed enhanced their intestinal proteome responses and self-defense against oxidative cellular level stresses [55]. Macroalgae-related proteomic aquaculture is still a growing field, and algae farming using proteomic techniques needs further exploration in the future. Recently, a nonanimal emulsifier peptide isolated from a seaweed species, *Eucheuma denticulatum*, using proteomic techniques has gained interest [56]. Additionally, comparative proteomics used to determine the environmental stress responses of a seaweed, *Neoporphyra haitanensis*, demonstrated increased levels of late embryogenesis-abundant proteins for protein protection, and the involvement of multiple enzymes in various metabolic activities [57]. Similarly, combined metabolomic and proteomic approaches using microalgae can be beneficial in integrated fish polyculture systems [26]. A recent review on aquatic insects as a dietary source determined that many aquatic insects contain over 50% of proteins and various kinds of amino acids, with an average of 51.6% essential amino acid content [58]. This implies that proteomics can act as a tool for identifying new culturable species for aquaculture. Extensive proteomic studies have been conducted on the temperate commercial food fish species, such as codfish, Atlantic salmon, and rainbow trout [26,59,60], whereas proteomics was applied in the fish quality-related studies using target species such as seabream [61].

Stress levels in the culture medium have a significant effect on fish growth, biochemical composition, wellness, production, and the end profitability. Proteomic studies enabled the identification of metabolic changes in fish species as a response to stress conditions [62,63]. Similarly, analysis of stress in intensive shrimp farming practices can be linked with proteomic studies to develop a stress-free culture medium. For example, a diverse range of protein profiles has been identified after stress exposure in the culture medium of Chinese shrimp, *Fenneropenaeus chinensis* [64]. Proteomic studies on the Pacific geoduck clams found the species to be highly resistant to acidified conditions, changes in temperature, and dissolved oxygen levels in water [65,66]. This indicates that proteomic strategy in aquaculture is a powerful technique that can be used to identify resistant species suitable for farming under variable natural conditions. Identifying stress responses in the culture medium would enhance farming conditions and the welfare of fish by reducing stress levels. Proteomics, physiology, and developmental biology methods can be applied to analyze the impact of contaminants on fish using a model or nonmodel fish species [67].

Aquaculture and fisheries-related food safety are imperative to ensure the consumption of healthy aquatic foods. The impacts of aquatic food safety and status are widely studied [68,69]. Various proteomic applications have been used to identify functional changes in aquatic proteins with nutritional interest, detect accurate labeling of seafood products, develop biomarkers for quality, freshness, pathogens, allergenic proteins, and seafood hazards and to evaluate the expression of recombinant proteins [48]. The European Union food safety regulations ensure the traceability and accurate identification of fish species to avoid seafood safety-related frauds, and proteomics can act as a novel strategy to distinguish commercially available fish food sources and their sex differentiation [48,70,71]. Microalgae-related proteomics mainly focuses on food safety by analyzing their responses to environmental stressors or pollutants [72]. Moreover, waste from aquatic foods has been identified as a source of biological hazards (pathogenic bacteria, biogenic amines, viruses, and parasites) and chemical/abiotic hazards (antimicrobial, formaldehydes, heavy metals, and microplastics) with potential health risks, and novel proteomics approaches can be used to analyze most of these risks.

A recent study revealed the toxicological effects of microplastics on *Litopenaeus vannamei* using proteomics and metabolomics methods [73]. Ingestion of microplastics poses health risks to aquatic organisms and can be harmful to the intestinal microbiota of host organisms. Nanoparticle contamination has been identified as an emerging abiotic hazard in aquaculture production systems, and targeted proteomics can ensure seafood safety from such hazards [52]. A comparative study on the effect of ionic and nanoparticulate silver (Ag) on the growth rate of microorganisms revealed that Ag ions strongly enhanced the growth rate of *Pseudomonas* spp., favoring biotic hazards [74].

For biotic stress studies, proteomic technology can be used to isolate and grow hazardous microorganisms in a culture medium or to directly identify them in fish products [52]. Concerning the clinical diagnosis and antibiotic resistance capacity, targeted proteomics has been used to identify proteins associated with bacterial infection [75]. A previous report applied a protein interaction map as a tool for identifying potential white spot syndrome virus treatment in cultured shrimp [76]. Proteomic-based harmful algal blooms have been applied to differentiate toxic from nontoxic dinoflagellates [77]. Additionally, antibiotic resistance-related proteomic studies have been intensively studied in aquaculture systems. Consequently, antibiotic resistance has been reported in finfish and shellfish-related microorganisms, suggesting economic loss in farming systems. For example, proteomics was used to confirm tetracycline resistance in *Aeromonas hydrophila* [52]. Similarly, the antibacterial resistance of *A.* *hydrophila* was determined in bacteria isolated from water and meat samples [78]. A proteomic approach was also used to detect the multidrug resistance of an *A. hydrophila* strain and the different regulatory mechanisms of proteins [79]. Antibiotic resistance of *Edwardsiella tarda*, an important infection in the seafood industry, was analyzed using a quantitative proteomic approach, and microbial protein regulation was determined during biofilm formation [80]. Proteomics strategies can also be enhanced or expanded to control antibiotic-resistant pathogens in aquaculture systems [81].

**Figure 2:**
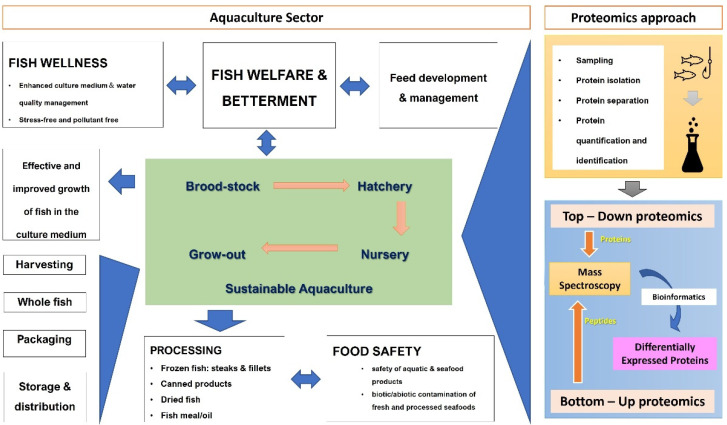
Overview of proteomic applications in the aquaculture sector and seafood industry. Fish wellness and betterment is highly important in the sustainable aquaculture practices. Proteomics techniques can be applied to monitor and manage culture environments and fish feed development. After harvesting, fish processing and food safety can be advanced by proteomics.

Seafood allergy testing is one way of applying proteomics in an aquatic system. However, new protein-based biosensors are needed to improve the identification, detection, and quantification of seafood allergens [52]. Targeted proteomics was used as a rapid assessment tool to identify β-parvalbumin, a key allergen of fish [82]. Proteomic profiling of tropomyosin, which is a major allergen of shellfish species (mollusks and shrimps), was conducted to characterize its complete amino acid sequence [83]. Figure 2 presents a summarized overview of approaches to proteomic application toward sustainable aquaculture and aquatic or seafood safety.

### 4.2. Proteomics in Aquatic Environmental Pollution and Monitoring

Genome and environment are the two key factors that determine changes in the proteome of an organism. Proteins cause biochemical and functional changes in organisms as a response to environmental changes [84]. However, limited and incomplete genome sequences are still a barrier to identifying proteins for ecotoxicological studies on aquatic life [38]. Toxicity in the aquatic environment is a source of stress to aquatic life, and their stress responses can be detected and measured using proteomics. For example, the accumulation of human and veterinary active pharmaceutical ingredients, which alter aquatic phytoplankton protein profiles, has been widely examined using novel proteomics technologies [85].

Ecotoxicoproteomics is a trending study area that was developed approximately two decades ago, with the application of a 2D polyacrylamide gel electrophoresis, and to date, targeted proteins have been quantified using the selected reaction monitoring (SRM) method, which is a powerful tool for analyzing predetermined proteins across different samples; more details on this technique are reviewed elsewhere [86]. The toxicity of engineered nanomaterials (ENMs) has been identified in aquatic bivalves, and the materials can cross the cell membrane barriers and disrupt the intracellular environment, leading to DNA damage [87]. However, damages caused by ENMs in aquatic environments are yet to be fully investigated. The application of new technologies such as SRM has been tested in freshwater *Grammarus fossarum* for multi-biomarker development [27]. These novel approaches can be used in ecotoxicoproteomics to facilitate advanced and reliable aquatic environmental monitoring; however, the techniques still need further development to study multiple aquatic organisms. Aquatic environment monitoring is one of the key strategies for aquatic conservation and management. Naturally available and measurable indicators or biomarkers are conveniently used in environmental monitoring, and novel biomarkers are widely searched in aquatic environments, such as oceans. However, significant ocean floor area must still be explored to discover new proteins, and their biomarkers properties must be confirmed by standards. Proteoforms are a novel characteristic approach to investigating stress-biological responses in the aquatic environment. Moreover, protein isoforms could apply to identify changes in the biological processes at the cellular enzymatic and transcriptomic levels.

Next-generation proteomics using protein databases is a reliable tool for discovering biomarkers such as conserved and ubiquitous proteins. The previous absence of protein databases was a major challenge [88]; however, various protein databases have currently been developed, and proteomic techniques can now be used to identify novel biomarkers in aquatic pollution studies. Protein isoforms, post-translational modifications, and protein interactions are associated with chemical pollution in aquatic environments [89]. A recent study showed that silver nanoparticles could affect protein folding, transmembrane transport, and translation [74]. The application of environmental proteins (eProteins) in environmental monitoring is a novel concept with potential use as biomarkers. Some eProteins are environmentally stable and their fate in the ecosystem is unknown. Subsequently, a study performed using a genetically engineered (GE) plant, GE Bt maize, to determine the fate of eProteins showed that the derived Cry proteins from the plants were pseudo persistent compounds from the nearby water systems, which suggested that Cry protein detection in the water system is due to the steady state of eProteins in the natural environment [90].

Plastic pollution is a severe threat to aquatic environments. Microplastics and nanoplastics highly contribute to the altered biological functions of aquatic life and have caused numerous environmental implications, such as the decreased photosynthetic ability of phytoplankton, cell growth inhibition, and heteroaggregate formation [91]. However, the cellular or molecular mechanism of microplastic- and nanoplastic-mediated changes in the biological processes of various aquatic organisms must still be explored on a large scale. Hence, recent proteomics technologies have a huge potential for future studies on the metabolic pathways’ modifications, biodistribution, and bioaccumulation caused by plastic molecules in aquatic environments.

Microplastics in aquatic ecosystems produce chemicals that change the homeostasis, osmoregulation, nutrition, reproduction, and molting of aquatic organisms and can be detected using proteomics techniques [92]. For example, a recent study using integrated proteomics and other omics strategies investigated the toxic effect of microplastics on edible shrimp, *Litopenaeus vannamei*, and the results showed that microplastics could change the protein profile of the hemolymph as well as the expression of immune-related proteins [73]. This indicates that proteomic technology is ideal for studying the impacts of microplastics and nanoplastics on edible aquatic animals and their potential health consequences in humans.

### 4.3. Natural Aquatic Proteins and Marine-Derived Medicine

Searching marine products for drug development is a remarkably developing industry that is interlinked with scientific research, the economy, and technological advances. Natural marine products have been described as valuable biomimetics and multifunctional raw materials for various industrial and biomedical development [93]. Common marine biomaterials are polysaccharides, such as chitin, alginates, fucoidans, carrageenans, ulvans, and agar; structural proteins, including spongin, collagens, gelatin, keratin, conchiolin, and conchixes; and biominerals, which include corals and shells. Aquatic proteins are directly or indirectly involved in the production of these biomaterials. Therefore, discovering such valuable proteins will be essential for the development of the biomaterials-related industries mentioned above.

A deep understanding of protein structure and domain specificities is important for functional product development. Chitin is a widely studied chemical compound that produces chitooligosaccharides and N-acetyl-D-glucosamine with various applications in agriculture, medicine, food, and cosmetic industries. Aquatic-based chitins are mainly derived from the cuticles of crustaceans such as crabs and shrimps. Chitin shows thermostability at 260–360 °C, which is a valuable trait for various industries [93]. However, chitin features are species-specific and can be altered depending on the chitin-degrading enzyme, chitinase, of the producing organism. A recent study identified 11 chitinolytic enzymes from *Pseudoalteromonas flavipulchra* and predicted their protein domain architecture to understand the chitinolytic enzyme secretion [94].

Marine-derived medicine is a rapidly developing field of aquatic science with the future aiming to apply proteomic strategies to enhance the discovery of aquatic-based natural drug products for use in human disease treatments. Marine-based gold nanoparticles such as chitosan have been used as a delivery drug for the diagnosis and treatment of cancers [95]. However, more proteomic-based studies on the roles of chitosan in oncology are still needed to potentially develop diagnostic kits and facilitate anticancer drug delivery.

Marine collagen is another sustainable area with potential biomedical applications in tissue engineering [96]. However, novel applications need exploration using proteomics and other omics technologies to determine the success of marine collagen as a scaffolding material in the biomedical sector. Metabolic pathways of natural marine products with drug potentials have been key objectives of marine medicine [97]. Reported studies between 2018 and 2021 identified 68 unique and patented natural marine products, and the majority will be used in biomedical applications [98]. Hence, there is a potential of growing aquatic proteomics-related sectors in the future.

## 5. Challenges and Recommendations

We identified challenges and recommendations for the three main sectors of aquatic proteomic applications described in this review. Technically, as a basic step, the success and efficiency of protein extraction from aquatic organisms are highly dependent on suitable protocol selection. We highly recommend developing a universal, standard, simple, easy, economical, and efficient protein extraction protocol for aquatic life. Although this presents huge challenges, family-specific protein extraction protocols for aquatic species can be developed to overcome the above constraints. The challenge with comparing marine algae-based protein composition is due to the differences in applied analytical methods [99]. This further warrants the need for a common protocol for analyzing protein in aquatic organisms.

Quantification of complex protein hydrolases from aquatic organisms is another challenge in proteomics. Although gel- and MS-based techniques have successfully been applied in other organisms, novel methods for quantifying aquatic protein hydrolases using the existing methods seem relatively challenging because of their complexities. Moreover, most aquatic protein hydrolases are contaminated with nonprotein substances such as polysaccharides. Therefore, protein quantification results using regular methods should be confirmed and validated with more than one technique. Additionally, amino acid analysis is another reliable and novel approach for protein quantification [100]. As a recommendation, a 2D differential in-gel electrophoresis coupled with iTRAQ is a suitable approach for identifying and characterizing aquatic proteins [101]. LC–MS-based protein quantification has been used in aquatic proteomics, for example, in the biomedical research area. A study on water fleas, *Daphnia pulex* and *Daphnia longicephala,* identified 531 and 317 proteins, respectively, using LC–MS/MS-based proteome profiling, and the results indicated evidence for many numbers of protein coding regions in the *Daphnia* genome for the first-time [102]. Another recent study identified proteome profiles of marine and freshwater *Synechocystis* strains using the LC–MS technique [103]. Additionally, the TMT labeling approach can be a useful proteomic-based protein characterization method with ease of automation. A detailed review of quantitative multiplexed proteomic applications determined that the isobaric tag quantification was the most accurate method [104].

Complete genome data are still lacking in many aquatic organisms, thus limiting proteomic application in their studies. Genomic and transcriptomic data are crucial for the accurate determination of novel proteins from aquatic environments. Therefore, we recommend integrating proteomics and other omics data in the identification and fingerprinting of proteins from aquatic organisms. Aquatic proteomic studies can be integrated with genomic, transcriptomic, metabolomic, and metagenomic approaches. Such methods will generate reliable information for the potential identification of biomarkers and metabolic pathways associated with environmental stress responses in aquatic organisms. Moreover, the integration of multiomics techniques can elucidate the underlying biological interactions among organisms and between aquatic environments. Protein database development and their structure analysis are two main challenges in their comprehensive identification. The recently developed AlfaFold protein structure database is currently one of the most reliable tools for characterizing the structure of key proteins. However, a protein database system specifically generated for aquatic organisms is required to fully apply proteomic-based approaches in aquatic environmental studies. Furthermore, we recommend possible solutions to the challenges in the three major aquatic sectors that are discussed in this review (Table 1). Additionally, the knowledge gap of aquatic proteoforms and their complexity is a limitation in proteomics application in aquatic environmental studies.

## 6. Conclusions

**Figure 3:**
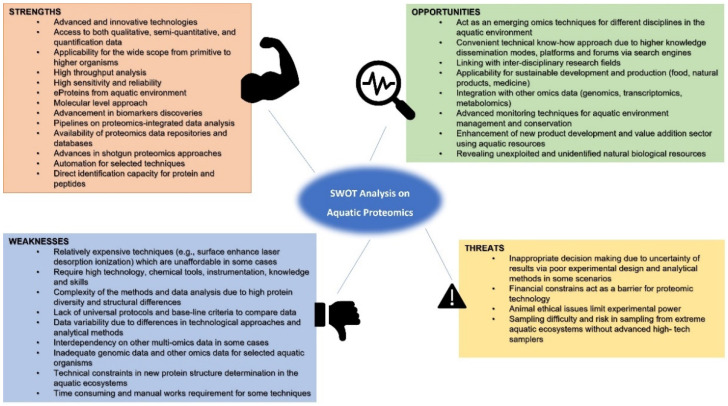
Strengths, weaknesses, opportunities, and threats analysis for aquatic proteomic studies.

The aquatic proteomics sector is vital to future developments in the aquatic sector. We identified strengths, weaknesses, opportunities, and threats to apply novel trajectories for successful proteomic applications in aquatic environmental studies (Figure 3). Proteomics in the aquatic environment has been applied mainly in the aquatic food sector, environment monitoring and management, and natural products identifications and developments. Under the food industry, proteomics can be mainly applied to enhance fish growth and wellness. The ELISA assay is one of the common practices of proteomics for testing seafood allergies. Ecotoxicoproteomics is a widely expanding area for monitoring the safety of aquatic environments and its stress on aquatic life. Some proteins are used as biomarkers to study environmental pollution and toxicity. Aquatic natural products are widely used in the biomedical sector. Novel proteins identification from the aquatic environment is opening many development opportunities to technologies as well as industries. However, difficulties in sampling and isolation of proteins are still major constrains for sluggish development of aquatic proteomics sector.

## Figures and Tables

**Table 1:** Common challenges and recommended solutions to proteomic applications in aquatic environment sectors.

Sector	Challenges	Recommendations
Food industry	Seafood safety and human health risks. Sustainable aquaculture development through modified feed, disease management, and pollution control. Quality of the aquatic product. Pathogens and biotoxins in aquatic food. Animal welfare and ethics.	Seafood allergies testing and ELISA assay development to check the quality of seafood with the application of proteomics. Enhancing aquatic feed quality and efficiency using alternative protein sources. Screening production with appropriate biomarkers as a diagnostic tool for infectious fish diseases in aquaculture farms. Use of macroalgae-based seaweed proteomics to identify novel aquaculture species. Sustainable aquatic, genetically modified food product development and conservation of edible aquatic animals using proteomic-based approaches. Application of advanced, high-throughput proteomic techniques for disease management and welfare monitoring.
Environmental monitoring	Monitoring pollutants and impact assessment. Conservation and management of aquatic life. Antibiotic resistance in the aquatic environment. Aquatic biodiversity assessment. Environmental toxicity.	Application of multiple biomarkers for aquatic toxicology studies. Species-specific proteomic database development for biomarker species. Developing global protein baseline data for the impacts of biological processes resulting from changes in the proteomes of aquatic organisms in response to environmental stresses. Explore unidentified novel species using proteomic data. Application of eProteins for biomonitoring and impact assessment.
Natural products	Novel natural product isolation and identification. Evaluation of biological activities. Drug development from natural aquatic products. Predicting protein domain architecture. Impact on biodiversity by harnessing. Nonanimal protein identification.	Estimation of biodiversity and interactions among organisms using proteomics in the study area prior to sampling. Extraction of minimum quantities from sensitive aquatic ecosystems as a conservation strategy. Implement bio or chemical synthesis processes. Develop and use high-tech equipment for underwater sampling. Use NMR spectroscopy to determine molecular structure. Seaweed proteomics for novel protein and peptide isolation.
